# Employees’ Resources, Demands and Health While Working from Home during COVID-19 Pandemic—A Qualitative Study in the Public Sector

**DOI:** 10.3390/ijerph20010411

**Published:** 2022-12-27

**Authors:** Laura Seinsche, Kristina Schubin, Jana Neumann, Holger Pfaff

**Affiliations:** Institute of Medical Sociology, Health Services Research and Rehabilitation Science (IMVR), Faculty of Human Sciences and Faculty of Medicine, University of Cologne, Eupener Str. 129, 50933 Cologne, Germany

**Keywords:** working from home, JD-R model, wellbeing, health, stressors, job resources, job demands, public service, qualitative data, COVID-19

## Abstract

(1) Background: The COVID-19 pandemic changed the working environment in Europe in March 2020, leading to an increase in working from home. In the German public sector, many employees experienced working from home for the first time. Despite the impact on employees’ daily working life, we know little about employees’ resources, demands and health while working from home. The aim of this study is to investigate how working from home is implemented in the public sector one year after the COVID-19 outbreak. In line with the job demand–resources model by Bakker and Demerouti (2007), potential resources, demands and health benefits of working from home are explored. (2) Methods: Semi-structured qualitative telephone interviews were conducted with twelve employees from different public sectors in Germany between December 2021 and February 2022. The semi-structured interviews were audio-recorded and transcribed verbatim, and the data was content-analyzed. (3) Results: Employees reported that personal resources, job autonomy, work task, collaboration, leadership, offers by the agency, work environment and equipment served as resources to buffer physical, social, psychological and organizational demands. (4) Conclusions: The research highlights job resources, job demands and potential health impacts of working from home in the public service. Furthermore, the study shows possible starting points for dealing with the health risks of working from home in the future.

## 1. Background and Current Research

### 1.1. Working from Home in the Public Service Sector

COVID-19’s preventive measures led to an extended use of working from home in Europe in 2020 [1]. Before 2020, working from home was common in France, Great Britain or Scandinavian countries in Europe, where employees worked from home on a regular basis [2]. In comparison to the European countries’ average, German companies offered less working from home and often work was carried out on an hourly basis instead of over full days spent working from home [2,3]. To define working from home during the pandemic, we use the definition of Bellmann and Hübler ([4], p. 424): “Working from home, also called remote work (RW), telecommuting, teleworking, homework, home office, mobile work, outwork and the flexible workplace, is a work arrangement, in which employees do not commute to their workplace in the company.”

In particular, in the public service sector working, from home was new for the most agencies in Germany [2,5]. According to Siegel et al. [5] only 2% of public service employees worked from home for more than 50% of their working time before the COVID-19 pandemic in Germany. During the first lockdown period this number increased to 45%, while 34% of employees worked 75–100% from home.

One reason for the low distribution of working from home before the pandemic was the lack of digital infrastructure in a majority of the public service agencies worldwide, and thus, employees were simply not enabled or allowed to work from home [6,7,8,9]. After the outbreak of the pandemic, the public sector reacted quickly to the changed work environment and offered working from home to employees to meet social distancing requirements [5]. In turn the pandemic increased the rate of digitalization in public service agencies because digital infrastructures were built in order to shift processes to the online work environment (e.g., [10,11,12,13]).

Additionally, there are other reasons for not introducing working from home earlier in Germany’s public service sector. These are rooted in the fear of a lack of social contact, unsuitable ergonomic work equipment and health risks when employees work from home. Work delimitation—due to a blurred private and business life—could lead to an increased workload and stress [14]. Another reason for the scarce prevalence of working from home in the public sector might be the attitude of supervisors and the work culture of the agency, which demands the physical presence of employees at the workplace in Germany [15]. Williamson et al. [16,17] reported similar findings in Australia and that the COVID-19 pandemic led to a change of managers’ allowance decisions regarding working from home.

Meta-analyses and systematic reviews have investigated outcomes of working from home worldwide (e.g., [18,19]). Studies regarding the work–life balance of public service employees have come to contradicting conclusions. On one hand, employees suffer from an increasing work–home conflict and work exhaustion [20], and on the other hand, working from home can increase affective wellbeing [21] and work satisfaction [22,23,24] and performance [25]. Other studies have shown that working from home can decrease stress [26,27]—as well as reducing stress associated with commuting [28]—and increase energy levels [27] and quality of life [24,28]. A study by Anderson et al. [21] linked affective wellbeing to social aspects in the work context; the findings showing that a high social connectedness, even when employees worked from home, reduced possible negative effects on wellbeing.

If employees can choose between working from home and working from the office, work–life balance is slightly higher for the employees that voluntarily want to work from the office. In comparison to employees that are not allowed to work from home, employees with the opportunity to work from home have a higher work–life balance [29].

Novianti and Roz [30] found no significant effect from working from home on work satisfaction. In the same regard, Garcia-Contreras et al. [31] found no link between job satisfaction and teleworking in their study on public servants in Mexico. Nevertheless, there were positive health impacts due to the increased freedom and flexibility in work tasks that led to a decreased level of burnout prevalence.

Studies have shown that many employees hope to work more hours from home in the future, even after the COVID-19 pandemic has passed [32,33]. This shows the need to explore the working conditions and work environment of public service employees in order to define suitable resources for working from home. Thus, the question is, how can the work environment be changed to an extended working from home culture that increases the health of employees? Therefore, the current study is grounded on the JD-R model by applying a qualitative approach and thus contributes to the state of research by identifying potential job resources and demands when working from home.

### 1.2. Job Demands–Resources Model (JD-R Model)

The JD-R model is a helpful construct for investigating the working conditions of public service employees. The JD-R model is grounded in several models such as the effort–reward imbalance model [34], the demand–control–support model [35,36], and the conservation of resources model [37]. It has been used in numerous studies (see, e.g., [38]) and in the context of telework or working from home (e.g., [39,40]).

The idea of the model is to explain how the organizational environment impacts performance and wellbeing of employees [41]. The model classifies the aspects of job characteristics in two categories: job demands and job resources [42].

Job demands are “physical, psychological, social, or organizational aspects of the job that require sustained physical and/or psychological (cognitive and emotional) effort or skills and are therefore associated with certain physiological and/or psychological costs” [42]. Examples include high work pressure, emotionally demanding interactions with clients or an unfavorable physical environment [42].

Job resources lie in aspects of the job that help reaching work goals, reduce job demands and the associated costs, and stimulate personal development and growth [43]. Examples of job resources are performance feedback [42]; autonomy, such as having the freedom to decide whether to work at home or the office [41]; and social support from your colleagues or career opportunities [42]. Additionally, personal resources were added to the JD-R model in a later version [41,42,44].

The combination of low job resources and high job demands can lead to serious health impacts such as burnout [41]. In order to prevent health impairments, organizations can optimize the work conditions by providing job resources and monitoring job demands [45,46,47].

### 1.3. Study Aims and Research Questions

Due to the COVID-19 development and increased working from home, we sought to get more knowledge about public service employees’ resources, demands and health while working from home. In the context of working from home, some job demands and job resources may be alternated, added or even lacking compared to working from the office. Using qualitative interviews, the present study strives to enrich the JD-R model with employees’ experiences in the public sector while working from home.

The study aims to answer the following research questions:

1. What are potential resources and demands when public service employees work from home?
2. In what way does working from home provide health benefits or impose health risks on public service employees?

## 2. Materials and Methods

### 2.1. Qualitative Study Design

We pursued an exploratory approach by choosing a qualitative design and gather in-depth insights to public service employees’ experiences of working from home [48]. According to Witzel and Reiter [49] problem-centered interviews offer the possibility to capture the social reality related to subjective perceptions, individual actions and ways of processing a certain topic with an unbiased approach. The exploratory approach was chosen because it is useful in applied health research when the topic is understudied [50,51,52]. Nevertheless, a systematic research process was ensured by following the literature on qualitative data analysis [53], quality criteria and reporting guidelines [54]. The study was approved by the Ethics Committee of the University of Cologne and prospectively registered (Ref No. 21-1417_1).

### 2.2. Sampling and Recruitment Strategy

For the present article, we used the existing contacts of a longitudinal survey study on working from home of public service employees. In the second wave of the survey, employees could provide their email address if they wanted to participate in the qualitative interviews. Since we obtained demographic details prior to the interview from the possible participants, we applied a purposeful sampling strategy [55]. Cases must be selected in such a way that findings related to the underlying question can be obtained with a high degree of probability [55]. If the selection is based on predefined criteria, this is also called criterion-oriented sampling. In this way, cases are identified that are similar or different in terms of the factors to be investigated. Heterogeneous sampling based on criterion-oriented case selection is particularly suitable for deriving theories or investigating the variability of the research object [56]. In this manner, we tried to obtain maximal variance in gender, age, leadership position and current job position. Other criteria were whether working from home was established in the agency before the outbreak of the COVID-19 pandemic and how well working from home was implemented from the participants’ point of view. There was one major exclusion criteria: participants that could not or only partially do their work at home due to technical limitations were excluded, since their jobs cannot be compared to other persons who have the option of fully switching to working from home. Additionally, two of the excluded participants reported a solely negative attitude towards working from home. We were careful with these potential participants, as they may have been negatively biased and did not fit into the sample. All the other participants were able to do their jobs from home completely. Unfortunately, some of the contacted interview partners did not participate in the data collection. In total, we contacted 20 persons from which 5 did not respond to our recruitment attempts. Thus, there is less variety in the final sample than previously anticipated. The participants were contacted via email with an invitation to the telephone interview. They received up to three reminders, if they had not responded to the first or following emails, which is in line with the tailored design method by Dillman et al. [57]. Three interview partners, who had agreed on a meeting, dropped out before the interview took place due to health issues or family reasons.

### 2.3. Study Participants

A total of 12 public service employees (mean age = 54.3 years; range = 29–62 years; 33% female) from different public sectors in Germany were interviewed. Employees’ length of work experience in the current agency lasted from 1 to 36 years (mean = 14.8 years). The minority of the participants had worked from home before the COVID-19 pandemic (33.3%). On average, they worked 2.25 days from home, whereas at time of writing, five participants worked from home on all workdays. Additionally, all of them reported that they had a fixed workplace when they worked from home. Of the 12 participants, 7 (58.3%) had a leadership position. All the participants’ details can be found in Table 1.

### 2.4. Semi-Structured Interviews

The SPSS principle by Kruse [58] was used to develop semi-structured interview questions, as well as narrative impulse questions. The acronym SPSS in German language stands for four different steps of developing interview questions, namely collecting questions, checking them for adequacy, sorting and assembling the questions into narrative impulse questions, and questions maintaining the narrative flow. Three researchers (J.N., K.S., L.S.) developed the semi-structured interview guideline. At first, possible questions were brainstormed based on categories from a previous survey study on working from home in the German public sector [59]. Questions were also brainstormed based on the construct of job demands and resources in the JD-R Model. Secondly, the questions were reduced and optimized in order to meet the standards of qualitative research (e.g., openness, suggestiveness). At last, the questions were organized into four topics with a narrative impulse and sub-questions [60]. The final interview guideline with guiding questions and sub-questions can be found in Appendix A.

Three female researchers (J.N., K.S., L.S.) who were trained and familiar with qualitative research projects conducted 12 semi-structured qualitative interviews. Written consent was obtained prior to each telephone call as required by German data protection laws. The interviewees supplied us with their signed consent forms via e-mail. Since social restrictions were present due to the COVID-19 pandemic, interviews were carried out over telephone. All the interviews were audiotaped and lasted between 26 and 60 min. Data gathering took place from December 2021 to February 2022. Each interview began with an introduction, followed by information on the study and its confidentiality, and a warm-up question. The interview then delved deeper into topics relevant to the research with the help of the semi-structured guideline. The questions were structured around four main topics: work organization, scope of action, leadership and collaboration and health. Each topic was opened with a narrative impulse question to encourage the interviewees in presenting their own experiences without the researchers’ implicit research interests interfering. The developed interview questions provided a flexible orientation framework that ensured that the main research topics were discussed and guaranteed a certain comparability between the interviews [49,55]. The order of the questions asked remained flexible, however, so that the interviewees were provided enough freedom to include their own topics and a natural narrative flow was maintained. The participants were encouraged to reflect on a typical day when working from home and their experiences. After the interviews, additional questions (e.g., number of employees, years of employment) were asked to complete an overview of the sample characteristics.

### 2.5. Data Analysis

The audio recordings of the interviews were transcribed according to the transcription rules outlined by Dresing and Pehl [61]. An external transcription service transcribed the interviews verbatim and pseudonymized all obtained data during the process. The qualitative content analysis method by Kuckartz [53] allows for the combination of a deductive and inductive approach. Analysis of the data was carried out using the software MAXQDA 2022 (VERBI GmbH, Berlin, Germany) [62]. Based on the JD-R model and the research questions, deductive categories were developed. The main developed codes included job resources (e.g., job autonomy, collaboration, personal resources) and job demands (e.g., physical demands, psychological demands, social demands).

In the next step, inductive categories based on the material were coded. They were partially based on new themes that the interview participants brought up during the interviews. Two researchers (K.S., L.S.) coded all of the interview material. After the first coding round, the results were discussed in order to develop a universal category system. In a second coding round, the second author (K.S.) examined whether the resulting coding scheme was applicable to the interviews. The final coding framework was discussed (K.S., L.S.), and data saturation was confirmed [63]. Afterwards, all transcripts were coded by the first author by applying the final coding scheme, which encompasses dimensions (research questions), main categories, and subcategories. The final coding system (see Supplementary Materials, Table S2: Final categories and quotes) includes representative quotations for each code for the purpose of inter- and intra-rater reliability [60]. The first author (L.S.) translated the quotations from German to English. For ensuring the quality of the qualitative research process, the completed COREQ checklist is available in the Supplementary Materials (Table S1: COREQ checklist) [54].

## 3. Results

**Figure 1:**
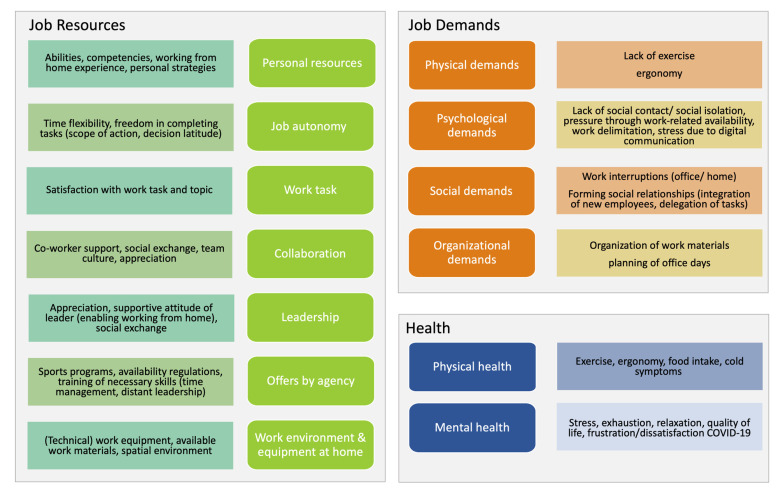
Main- and subcategories of the conducted interviews.

In the following section, the results of the conducted interviews are described in detail. Figure 1 shows the main categories and subcategories imbedded in the JD-R model.

### 3.1. Job Resources of Public Service Employees

#### 3.1.1. Personal Resources

In this category, all personal resources were coded, e.g., abilities and competencies that helped employees to cope with the situation when working from home. Furthermore, the codes involve experiences and strategies used to develop own work routines when working from home.

The ability to learn and develop new skills, such as the handling of software that is required when working from home, supported employees in using it effectively.

“So I’m very fortunate, I’ll say, in the use of my electronic tools, the programs that I have available and the software and so on, that I get on well with them and that I try to use them effectively for myself.”

(Interview #1)

One employee stated that he had to learn self-discipline in order to structure his workday:

“And that works quite well in most cases now. So this self-discipline is simply very much necessary, and I had to learn that myself first.”

(Interview #8)

The prior experience of working from home before rapid changes due to the COVID-19 outbreak might have helped employees to feel secure when working from home because they had already built up the necessary competencies. For example, one of the employees with a leadership position stated that for employees in their agency, working from home was a “routine operation” (interview #7), since they had already built up 10–13 years of experience working from home.

#### 3.1.2. Job Autonomy

Autonomy refers to the freedom experienced that employees rely on when fulfilling their tasks at home.

They were mostly free to organize their workday according to their own rhythm:

“I think that’s the higher self-determination. So I can, for example, what’s a very big factor is taking breaks. So I really have a problem taking a break here in my office.”

(Interview #3)

The same employee explained that she tended to go beyond her own limits when she worked in presence at the office, while she followed her own needs at home. Another employee stated that being able to vary his work output is

“one of the advantages of working from home”.

(Interview #7)

They enjoyed their decisional latitude regarding the choice of clothing:

“The most obvious freedom is, of course, that of clothing. Of course, since we don’t have any video calls with high-ranking ministers or anything like that (laughs), we all sit in front of the screen in our leisure jogging gear, I’d say, and that’s just pleasant, you don’t have to pay that much attention to it now, do you?”

(Interview #6)

Another gain from public service employees’ perspectives was how flexibly they could handle time when working from home. This means they could assemble and split their tasks during the day in order to have a longer lunch break and combine it with personal activities (e.g., interview #6, interview #10). Additionally, they gained more time because commuting was not necessary.

“The additional flexibility gained (..), but essentially limited to the fact that there was simply more time available due to the elimination of commuting.”

(Interview #8)

#### 3.1.3. Work Task

The work task itself can be a resource for employees if they enjoy working on the given topics (e.g., interview #1, interview #10).

Concerning working from home, one of the employees stated that she could find positive aspects of working on tasks differently at home, even though she missed working in the presence of others:

“Apart from that, of course, you can sometimes enjoy sitting at your desk and working on a translation with a lot of fine-tuning and in between doing a bit of terminology work, i.e., extracting terminology from the texts, etc. But it’s often a bit much computer work for me.”

(Interview #5)

#### 3.1.4. Collaboration

In this category, all aspects of working together were coded: Support between employees, social exchange, team culture and appreciation.

One interview partner stated that “the team spirit is certainly not as strong as it was before” (interview #7), while another reported that team spirit and collaboration did not change due to extensive working from home:

“It works quite well, so we don’t notice any loss of performance or friction in the form of operational failures. But it’s just a different way of working.”

(Interview #6)

Furthermore, one employee enjoyed the “ability to use technology” and to start a video call “at the push of a button” in order to speak with a colleague or ask for help (interview #12). In some cases, the employees from the IT department visited their colleagues at home to support them, when they first set up their technical equipment for working from home (interview #4).

A resource that was highly missed by employees was the personal contact and opportunity for a quick chat that was a given when working at the office.

“These are the things that are missing, or colleagues from other areas that you meet and talk to, or that you arrange to meet for lunch, or things like that. These things are missing.”

(Interview #10)

They missed the “unplanned communication” (interview #8) and the forming of “new ideas” (interview #2), which only occurred when all of them worked together at one place. This longing turned to joy when they finally got together and saw each other (e.g., interview #9). The proximity to colleagues at the office makes it possible to ask for help, if needed. Regarding digital communication, more inhibitions to contact colleagues exist when one works from home:

“…and then when they do call, they even apologize for calling “Sorry to bother you,” where I then think: Yes, but I’m on duty. If I was sitting in the room next to you right now, they would also come in and just ask.”

(Interview #3)

Regarding the quality of communication, different opinions were voiced. For some the quality and “intensity” of interaction was lower (interview #10), whereas others stated that collaboration profits from the reduction of communication on a factual level:

“So except for the communication thing a little more direct, straighter. By not leaving things and waiting until you can look for someone, but doing it immediately, then you have it out of your head. That’s what (four? many?) colleagues did and I thought that was a real improvement.”

(Interview #7)

Other positive resources in this section include received feedback and appreciation from coworkers.

“But as a rule they are all very positive, not just towards me (laughs very lightly), I think, but it’s just such good practice that most people say thank you when you’ve done something for them and at conferences or interpreting appointments, of whatever kind, this of course comes into play even more, because the participants in these conferences also notice how difficult it often is with the sound quality at such events.”

(Interview #5)

#### 3.1.5. Leadership

Leadership is experienced as a resource if supervisors have a positive attitude towards working from home and support the implementation of working from home. For example, one supervisor told her employees that they could take their work equipment from the office to set up their workplace at home, even though it was not common practice at that time:

“I think that was not allowed. I don’t know, by now it might be allowed, I don’t know. Anyway, when working from home started due to Corona, I told them to “Take the monitors home” so that they can work properly.”

(Interview #2)

Trust is essential when it comes to distant leadership (e.g., interview #6, interview #11). Some employees reported that it was difficult for leaders to adapt to the new situation at first, but the shared learning experience helped to bridge that gap:

“(…) there is no kind of social control or somehow/because everyone does it, it’s the same situation for everyone, so no one says, “Yes, you sit at home and put your feet up,” right?”

(Interview #6)

One of the supervisors explained how working from home transformed his attitude towards a work culture that is based on work results instead of presence at the office (interview #11). In a similar manner, interviewee #7, who also had a leadership position, stated that there is “no better control through presence at the workplace”. Regarding a regular exchange when working from home, one employee experienced that his supervisor “tries to talk to each of us at least once a day (…)” (interview #6), whereas other employees seldom had contact to their supervisor (e.g., interview #11).

Another aspect is the appreciation that employees receive from their supervisors for their work. It functioned as a resource but was a burden if missing:

“It works, it works well. So you also receive praise or whatever, or personal interest, and I can’t complain about that this year. But it has also been different. With other superiors before that, it was totally different, and that’s something that was also a burden for me.”

(Interview #1)

One of the supervisors even stated that employee appraisal was easier over online communication because nobody else could see who was in his office or for how long (interview #12).

#### 3.1.6. Offers by the Agency

The agencies had different means of supporting public service employees when working from home. For example, they offered leisure hours that can be used for sports activities in order to ensure that employees performed enough exercise at home (interview #2). Online diet (interview #6) and online fitness programs were also mentioned:

“(…) we have relatively many offers from the [name of agency], like so, there are (..) lunch break impulses or somehow from [name of agency], also active lunch breaks. I have not used all of those at all, but I could imagine that perhaps this has also helped other employees a bit, yes not necessarily structuring, but perhaps that thereby contact was also once again established to other people.”

(Interview #9)

Other measures included more participation in choices regarding purchased work equipment (interview #5) and monetary grants for the purchase of work equipment (interview #6). One of the supervisors reported that his agency provides online training of required skills such as distant leadership (interview #8), while another described the training department as “Leading at a distance, that’s new, they don’t know that.” (Interview #11).

#### 3.1.7. Work Environment and (Technical) Equipment at Home

Public service employees could set up their workplace at home according to their wishes and needs. They profited from separate office rooms at home or using their own equipment:

“And that is also sacred to me. I set that up for myself when I first started telecommuting, which was early 2019. It’s a room of its own. I built a huge desk myself that goes through the entire length of the room. Then I have a private workstation there, my official IT workstation. Here I also have absolute peace and quiet, and I enjoy that. And I can close the room. So then, I can’t see what’s there.”

(Interview #11)

The work environment at home could be a substantial resource when other family members did not disturb during working hours (social demands) and the living situation allowed, for example, to spend time in one’s own garden (interview #12). The public service employees chose to use additional private equipment such as larger or more computer monitors in some cases (e.g., interview #2, interview #1).

### 3.2. Job Demands of Public Service Employees

#### 3.2.1. Physical Demands

Some physical demands were omitted due to working from home such as commuting (e.g., interview #4) or business trips (e.g., interview #3), which involve long sitting or standing activities.

Simultaneously, the lack of commuting with public transport or even riding a bike to work resulted in a much lower physical activity level when employees worked from home.

“ (…) when I notice that I’m a bit sedentary, because I don’t have to travel to the bus stop in the morning, I don’t have to walk from the bus stop to the workplace, so those are all steps that are no longer necessary.”

(Interview #9)

Therefore, employees sat at their desks for long hours since the online meetings sometimes had no room for breaks (interview #8). If the used work equipment at home was not ergonomically suitable, it resulted in physical pain in some cases:

“In the beginning, […] I kind of worked at the kitchen table, and then I got really bad back pain, but I’ve had a standing desk for four months now, and since then it’s been okay again.”

(Interview #9)

#### 3.2.2. Psychological Demands

One of the psychological demands was the lack of social contact that public service employees experienced when they worked from home. They missed the interaction with other colleagues and small talk, and even felt cut off from information when they were isolated:

“If one has not seen then some colleagues perhaps (about a half year?) and then you start to notice the first impacts, where one has overheard things that a colleague has quit or has retired, the other has changed also the ministry, has quit or has oriented himself elsewhere...”

(Interview #6)

Other demands were the constant alignment of work and private life in order to reduce work delimitation (interview #1). The work-related availability and experienced pressure to be available for supervisors and colleagues played a large role.

“I think it’s more that I put myself under a bit of pressure, that I want to be available all the time (laughs lightly) because I want to show that I’m doing well. But I don’t think the superiors expect that at all, yes.”

(Interview #9)

Another aspect was the expectation of colleagues and supervisors regarding availability:

“There is now an expectation that you are always there [available on the phone] from 8 a.m. to 6 p.m., as it were.”

(Interview #9)

These expectations led to experienced stress and pressure and enhanced work delimitation, since public service employees worked overtime or just did not find an end to their working day:

“(…) and in the evening also to find an end, because by this sluggish beginning I have determined personally for me, it is very tempting to turn on the computer just this one time or in the evening at 10 o’clock.”

(interview #8)

#### 3.2.3. Social Demands

The employees stated different social demands whether they worked from the office or from home. In the office, the small talk could be unwanted and “there are always situations where you can’t escape as quickly” (interview #12) or coworkers could disturb the ongoing work process with their questions:

“Then the next colleague comes and says “Someone just called. I couldn’t tell you exactly either. Can you help me?” So then you’re always out of your processes.”

(Interview #3)

At home, family members could interrupt work (interview #5), or the living situation was so cramped that online meetings took place in unsuitable places such as the living room (interview #2).

Furthermore, working from home made it difficult to integrate new employees into working processes and as team members (interview #8) or for supervisors to delegate tasks (interview #10). In total, the building of relationships and ensuring relationship quality was more difficult:

“Small talk should not be underestimated. It’s also important for the relationship.”

(Interview #3)

#### 3.2.4. Organizational Demands

Working from home imposed different organizational demands on public service employees than working at the office. At first, employees had to organize the materials that they needed for work.

“Yes, of course, when someone gets the notebook home and then has to look at a 14-inch monitor all the time, I think that wears you out. No, I’m quite sure of it: It gets on your nerves, and it also wears out your performance.”

(Interview #12)

They may have taken necessary work equipment and materials home in order to fulfill their tasks. Having technical equipment at home became a burden if it was not supplied or not working properly. Particularly at the beginning of the working from home period, resources were limited:

“We had some laptops, but not everyone, so that at the beginning the laptops had to be exchanged in part. So one person got the laptop for a week and the other had to find something else to do at home, which was difficult.”

(Interview #11)

Other challenges were that not all information was provided online (e.g., interview #3, interview #8), monitors were too small (interview #12), and printers were missing (interview #2).

Additionally, employees had to plan office days according to their tasks:

“There are a few tasks that you can only do in the office. For example, signing invoices and the like, which we don’t yet do electronically. But then we also arrange to meet sometimes.”

(Interview #5)

Public service employees scheduled tasks that are performed best from the office on their planned office days and even organized to meet colleagues on these special days.

### 3.3. Health Outcomes

#### 3.3.1. Physical Health

Regarding their physical wellbeing and health, public service employees reported mixed results. For some, exercise was missing, while others felt that they had more opportunity to integrate their sports activities during the day:

“Because I simply do some exercise at lunchtime and I somehow have the feeling that after lunch or after a lunch break, my brain switches off automatically, which is a bit of an exaggeration, but when I work from home I really start somehow fresher into the second half of the work day after lunch break.”

(Interview #9)

Another employee reported that “exercise is missing [and] the physical tension increases (…)” (interview #8). Health impairments such as back pain were mentioned due to physical tension or the lack of ergonomic work equipment (e.g., interview #6).

Regarding food intake, the public service employees reported changed eating habits, which from their point of view transformed for better or worse.

“So at lunchtime, that one cooks for oneself or cooks in larger communities, that has with the contact ban to do, happens now rather not. So meals tend to take place in isolation. That’s stupid. That’s really stupid, isn’t it? You have to say that so clearly. That’s a cut, and then at some point n-tv doesn’t help anymore if I watch the latest news (laughs slightly) while eating, which is also totally unhealthy.”

(Interview #12)

“(…) because business travel diet consists of curried sausage and hamburger and, yes, serving fries or something like that in the hotel. But now when you’re at home like that, you can make yourself a cauliflower soup and make yourself a salad or something like that and eat, I think, healthier.”

(Interview #2)

Lastly, the COVID-19 prevention measures had another positive effect on health, because employees experienced fewer cold symptoms.

“(…) that someone coughs on you or something, that all falls away. So I’m actually/no, I haven’t had a cold at all during all this time.”

(Interview #5)

#### 3.3.2. Mental Health

Concerning mental health, different subcategories such as stress, relaxation, exhaustion, social contact, life satisfaction and frustration due to COVID-19 were formed.

In total, employees reported positive impacts on their health in general, because they experienced less stress, which was partly due to the saved commuting time:

“Not that I would feel mentally ill now, but that’s just this stress factor, you know? This feeling of stress is much lower when I work from home. This stress factor. And that overall, of course, has a positive effect on my overall health situation.”

(Interview #3)

“Because for the colleagues the stress, the stress of commuting and the stress of being annoyed every morning about train things and the devil knows what all, the long travel time or traffic jam or whatever, falls away. And nobody can tell me that standing in a traffic jam for hours in the morning isn’t stressful.”

(Interview #7)

In the same regard, one interviewee explained that he experienced less exhaustion since working from home.

“I think I feel much fitter since I started working from home. I mean mentally. So that means this exhaustion that I sometimes had when I was at work [at the agency] for a long time.”

(Interview #11)

For one employee, the chance for relaxation was also higher when she worked from home because she “can better take breaks, relax better in between or maybe even go for a walk around the block” (Interview #3) than when she worked on site. Positive views regarding experienced quality of life:

“The fact that I can organize my everyday life so flexibly means that I no longer have to travel to work. So I have a travel time of over three hours in total (…). I would say yes, totally. So I have more quality of life because of it, much more time to do things that would otherwise simply be not feasible.”

(Interview #9)

Negative aspects that were voiced concerning mental health often considered the COVID-19 pandemic and were not only linked to working from home. Social distancing measures impacted mental health of employees during COVID-19 as well because, e.g., their sports clubs were closed and their social contacts were abandoned:

“But I know from others who have a lot to chew on, the social contacts, in between just being able to laugh with others or the like. That puts a damper on the mood. Fortunately, that’s not the case for me personally.”

(Interview #8)

The ongoing COVID-19 pandemic caused frustration and could lead to “a cabin fever” and “a certain dissatisfaction” after some time (interview #10).

## 4. Discussion

### 4.1. Main Findings

The aim of our study was to acquire more knowledge about public service employees’ resources, demands and health while working from home. In this case, our study contributes to the existing research on working from home in several ways. First, we used the JD-R model [42,43], identifying potential resources and demands when employees work from home, adding to the existing theory.

Second, our findings contribute new or altered job demands and resources in the context of working from home during the COVID-19 pandemic.

Third, we discuss findings of the existing literature on working from home with our findings to guide future research and practice in providing health benefits or preventing health risks for working from home. Our research questions were the following:

1. What are the potential resources and demands when public service employees work from home?

Employees reported that personal resources, job autonomy, work task, collaboration, leadership, offers by the agency, work environment and (technical) equipment served as resources to buffer physical, social, psychological and organizational demands. In the following discussion, the aspects of the research that can be linked to other findings are highlighted.

Due to the shift of work processes to the home environment, resources and demands are altered, added or even lacking. For example, new resources, or rather deepened resources, are job autonomy and granted time flexibility. The findings fit the key advantages and disadvantages of working from home, which Ipsen et al. [64] reported for knowledge workers of 29 European countries. The main advantages were improved work efficiency, greater work control and improved work–life balance.

One of the game-changers in terms of positive or negative experiences when working from home is the personal work environment at home. If it lacks technical equipment or ergonomic equipment, employees have to face organizational demands and possibly buy suitable equipment themselves. Some employees enjoy setting up their own workstation at home and using private equipment that might further meet their needs. According to Ipsen et al. [64], inadequate tools were reported as one of the main disadvantages.

Other resources that become more important are personal resources and distant leadership. Leadership can be seen as both a resource and a demand depending on the leadership style and distant leadership skills of the supervisor. If the leader demands presence and the employees feel obligated to show extended work-related availability, it imposes psychological stress. If the leader, on the contrary, supports employees working from home and ensures a regular communication, leadership can be seen as a resource. Tummers and Bakker [41] recently discussed the classification of leadership in the JD-R model, but they arrived at the conclusion that leadership influences the job demands and resources that employees experience. From our findings, similar results point out that leaders can shape the job resources and demands (e.g., by providing employees with the right equipment), while the attitude of the leader might act as a demand or resource in itself. As in the findings of Williamson et al. [16,17], the attitude of supervisors plays a large role in managers’ decisions to allow work from home. The public service employees stated that after one year of extended work from home, they perceived a change of the presence culture that their supervisors facilitated prior to working from home.

An agency can provide offers that function as a resource for employees who work from home such as online coffee breaks or online exercise courses. Personal resources such as skills can help to manage the workday from home. Employees may also profit from prior experience working from home. Bezzina et al. [65], who investigated Malta’s public service, found out that working from home experience supported public servants in changing to remote work as well.

Job resources that have been altered include collaboration (changed to the digital work environment) and in some cases the work task (e.g., no business trips).

Furthermore, the findings concerning social contact are contradictory. For one, employees enjoy less interruptions in their work processes when working from home, but they miss the opportunity of personal contact at the office. The resource of personal contact is highly missed by them. In the same regard, Bezzina et al. [65] stated that Maltese public service employees experienced higher levels of productivity, but that they also felt secluded with the lacking presence of their coworkers when working from home.

New job demands have formed, such as dealing with work delimitation and separating work and private life. In this matter dealing with expectations regarding work-related availability is a psychological demand. Another organizational demand specific to a working from home setting is the planning of office days and scheduling of certain tasks according to the available work equipment.

Social job demands that have been altered are interruptions during work. Colleagues might not disturb work routines, but family members of employees can influence work productivity at home. One physical demand that is completely diminished is commuting.

2. In what way does working from home provide health benefits or impose health risks on public service employees?

If job demands are high and there are not enough job resources to balance out demands, potential negative health impacts can occur [42]. The findings of Sardeshmukh et al. [40] indicate that job demands (time pressure, role ambiguity and role conflict) and job resources (job autonomy, feedback and job support) mediate the relationship between telework on job engagement and exhaustion.

Concerning physical health risks, employees show highly sedentary behavior, since commuting and walking to the offices of other colleagues are omitted. This sedentary behavior, sometimes even combined with a lack of ergonomic work equipment at home, imposes risks. Similarly, Xiao et al. [66] showed decreased physical activity and exercise in their study, which took place during stay-at-home restrictions. Our findings indicate that exercise still needs to be planned or scheduled in employees’ daily lives, since commuting and associated exercise often fall short.

Diminished commuting has one important advantage: it can lead to reduced stress [28] and therefore to increased energy levels [27]. Additionally, employees suffered from less cold symptoms due to social distancing measures.

Employees reported mixed results concerning their mental health. Some experienced less stress, a gain in life satisfaction, more time for relaxation and less exhaustion, while others felt more stressed and suffered from the experienced isolation due to the lack of social contact.

Potential health benefits are linked to experienced job autonomy and time flexibility [31,40]. In a study by Garcia-Contreras et al. [31], increased freedom and flexibility in work tasks led to a decreased level of burnout prevalence. For the work–life balance of some employees, it is a good thing to integrate personal matters into the workday or combine work and family matters.

On the contrary, this behavior may become dangerous for employees’ health if work hours are extended or employees work after hours [14,20]. In this regard, extended work-related availability may impose health risks.

Other benefits based on the granted flexibility are the ability to assemble work according to one’s own needs and taking breaks flexibly. Since employees skip commuting, they save time that can be used for the preparation of fresh meals or exercise. Similarly, other studies report increased affective wellbeing [21] and quality of life [24,28].

Less interruptions from coworkers can lead to greater productivity when public service employees work from home. This may lead to increased work satisfaction. Furthermore, positive results for job satisfaction [22,23,24] and job performance [25] have been reported for employees working from home.

### 4.2. Strengths and Limitations

Some limitations need to be considered when interpreting our findings. One limitation is grounded in the selected sample of public service employees, because the experienced resources and demands may differ in other agencies and fields. There might have been a selection bias regarding the interview partners in this case, in that only interviewees who preferred working from home and are interested in the topic volunteered for the study. Most of the participants were over the age of 50, which needs to be taken into consideration when interpreting the findings, since job demands and resources may vary for other age groups. Another limitation is the subjectivity in interpretations that may differ from the intended interviewees’ perspective. Additionally, the interviews took place at a specific timepoint and only captured the views of public service employees working from home one year after the beginning of the COVID-19 pandemic.

A certain strength of the study is that three researchers participated in the study, conducted the interviews and coded the interview material—limiting the above-mentioned subjective bias. The qualitative reporting criteria and semi-structured interview guideline supported a standard procedure. Additionally, the research was grounded on the JD-R model [42,43], which is a widely used model, and contributes to the existing theory. Our findings contribute new or altered job demands and resources in the context of working from home during the COVID-19 pandemic.

To the best of our knowledge, this was the first study to explore the insights of public service employees regarding their working from home experience in the context of the COVID-19 pandemic in Germany. This qualitative study points out certain patterns and hints for future research to improve public employees’ working situations and health when working from home.

### 4.3. Implications for Research

Future research is needed, since the COVID-19 pandemic has transformed the work environment dramatically to working from home. The transferability of results to other groups of employees or even sectors should be investigated using quantitative methods. Therefore, larger samples and longitudinal designs could aim to gain a profound understanding of the relationships between job demands, job resources and their health impacts when employees work from home. Ideally, future studies will take place post-COVID-19.

Consequently, workplace health interventions and trainings aiming at public employees’ skills that enhance their wellbeing when working from home are needed. On particular, the success factors for distant leadership demand further investigation in the context of public service administration.

The results show that working from home offers great advantages that cannot be provided if employees are limited to the grounds of the office. Simultaneously, there are health risks that should be limited such as increasing psychological demand and stress due to the feeling of work-related availability or lack of social contact. Thus, hybrid work arrangements could be a possible solution. Future research should answer questions concerning hybrid work arrangements such as: How many days should employees be working from home and how many from the office? What is the ideal mixture?

### 4.4. Implications for Practice

For practitioners, the results show the importance of strengthening the skills of public service employees in order to organize days working from home. Time management skills and self-discipline are needed to work from home effectively. Additionally, leaders in public service have to be educated in distant leadership and build necessary skills.

In order to decrease the stress and pressure that come with work delimitation and work-related extended availability of employees, strict rules are needed. The agencies should establish a common agreement on the presence and work-related availability expectations of both employees and leaders.

Since decreased social contact opportunities led to public service employees feeling less informed and integrated in the organization, there is a greater need to create opportunities for social contact and gatherings. Moreover, agencies that established, e.g., shared desk policies during the pandemic, and want to continue this system in the future can profit from thinking about creating events strengthening their employees’ sense of community.

Agencies should provide job resources and decrease job demands where possible. From the results, the lack of (technical) equipment is named as one of the demands when public service employees work from home. If technical equipment and a suitable equipped workstation are provided, they function as a resource for the employees. Simply providing the necessary equipment—and training for, e.g., newly introduced software should have profound effects on employees’ wellbeing and health.

The reports of the interviewed public service employees show that not all employees want to work from home. Some employees do not have a suitable work environment at home; even if the technical equipment allows working from home, others simply prefer a strict separation of private and office life and thus the office to work.

It is important to provide employees the freedom to enjoy the flexibility that comes with working from home and the choice of whether or not to do so.

## 5. Conclusions

Overall, the findings of this study point out potential job resources and job demands of public service employees working from home. One of the resources is the granted time flexibility and autonomy, while handling extended work-related availability and work delimitation are mental demands reported by the interviewed public service employees. Despite the discussed limitations, the study shows possible starting points for dealing with working from home-related health risks in the future. Future research is needed to integrate the positive aspects of working from home and working in the office in order to find an ideal hybrid work arrangement. In practice, agencies need to support employees with regulations concerning working hours, work-related availability and training to foster the personal skills necessary for working from home.

## Figures and Tables

**Table 1:** Characteristics of the interviewed public service employees.

Interview	Age	Gender	Field of Agency	Number of Employees in Agency	Work Experience in Agency (Years)	Leadership Position	Start of WFH	WFH Amount (Days/Week)	Fixed Workplace (WFH)
**1**	59	m	Building and real estate	ca. 180	31	n	March 2020	5	y
**2**	61	f	Construction industry	4.000	28	y	2016	5	y
**3**	60	f	District governance	7 (in unit)	14	y	March 2020	0 (Before 4–5)	y
**4**	50	m	Data protection	ca. 175	3	y	before COVID-19	3	y
**5**	62	f	Social welfare	1.100	36	n	Spring 2020	ca. 4	y
**6**	53	m	IT service	300 (in department)	2	n	March 2020	5	y
**7**	62	m	Building and property management	2000	16–17	y	ca. 2009	1–3	y
**8**	49	m	Environmental management	1200	20	n	March 2020	3–4	y
**9**	29	f	Learning and education	1000	1	n	April 2021	4–5	y
**10**	56	m	Information and statistics	300	10	y	March 2020	5	y
**11**	55	m	Customs	10 (in unit)	12	y	2019	4	y
**12**	55	m	Telecommunication	3000	4	y	March 2020	5	y

Note: WFH = work from home.

## Data Availability

The data are not publicly available due to ethical and legal re- strictions, as participants of this study did not agree to their data being shared publicly.

## Supplementary Materials

The following supporting information can be downloaded at: https://www.mdpi.com/article/10.3390/ijerph20010411/s1, Table S1: COREQ checklist; Table S2: Final categories and quotes.

## Appendix A

**Table A1:** Interview guideline questions.

Guiding Questions	Sub-Questions
**Warm-Up Question**	
**Please tell me briefly how the introduction of WFH went in your agency.**	- Certain regulations on the part of the agency? - How were these coordinated? - Will WFH remain in the future?
**Organization of Work**	
**Please tell me what a typical day looks like, when you work from home.**	- Do you stick to a certain daily routine? - Have you found your own personal approach to the daily work routine when WFH? - When and how are you available for colleagues? - What has helped you to organize your work when WFH?
**In what way can you see differences to the way you work when you were present at the office?**	- Have you found that you work different hours than when you were present at the office? - Has your workload changed? - Unchanged/increased/decreased?
**Please tell me about the difficulties you experienced at the beginning when WFH.**	- What has improved this now? - What has helped you?
**Scope of Action**	
**Please tell me about the freedom you have in completing your work tasks when WFH.**	- What do you like about your work? (What motivates you?) - To what extent do you have time or performance constraints? - To what extent are mistakes tolerated? (Control) - How much responsibility are you given?
**To what extent do you notice differences compared to your freedom/scope for action in presence?**	- To what extent do you feel pressure to perform? (more/less) - Is the work more/less controlled? - ((work intensification?))
**Leadership and Collaboration**	
**Tell me how you perceive the interaction between managers and employees in your agency.**	- How often are you in contact/communication with your manager? - How is the communication with your manager in the home office? - Does the need for discussion come from you or does the manager approach you? - How do you experience recognition and appreciation at your workplace? (on the part of colleagues, on the part of the manager) - In what form does your manager express criticism? How do you deal with it? (work, person)
**To what extent can you identify differences in the way managers and employees deal with each other when they are present?**	- How does behavior transfer to the WFH situation?
***Example:* Do you have the feeling that there are colleagues in your organization who intentionally send emails very early or very late, which was not common before WFH?**	- Did you notice something in this way? - What do you think is the reason for this? (e.g., recognition performance, commitment)
**Health**	
**Please tell me how working from home affects your health.**	- Positive/negative? - Why is it so? - Physical problems (e.g., tension, fatigue, headaches) - Mental problems (balanced, full of energy vs. feeling anxious, distracted) - Life satisfaction?
**How was it for you in presence?** **(Can you notice any differences in your physical or mental health?)**	- Positive/negative? - Why is it so? - Physical problems (e.g., tension, fatigue, headaches) - Mental problems (balanced, full of energy vs. feeling anxious, distracted) - Life satisfaction? - If applicable, what is the biggest advantage of the home office for you?
**Case: You notice cold symptoms in yourself. Would you go to work?**	- How would you act when WFH? - How would you act when working in presence? - Why yes/Why not?
**Finally, is there anything you would like to add that you have not yet mentioned?**	
