# Supplementary material for: Employees’ Resources, Demands and Health While Working from Home during COVID-19 Pandemic—A Qualitative Study in the Public Sector
Source: Int J Environ Res Public Health. 2022 Dec 27;20(1):411. doi: 10.3390/ijerph20010411 (PMC9819647; doi:10.3390/ijerph20010411)
Supplement: Supplementary file 1 [file ijerph-20-00411-s001.zip › Table S2-Final_categories_and_quotes.pdf]

Theoretical Dimension	Main category	Sub-category	Definition	Quotes	Coding rules
Job Resources			Job resources refer to those physical, psychological, social, or organizational aspects of the job that are either/or:<br>-Functional in achieving work goals.<br>- Reduce job demands and the associated physiological and psychological costs.<br>-Stimulate personal growth, learning, and development. (Bakker & Demerouti, 2007)		
	Personal Resources		An important extension of the JD-R model is the inclusion of personal resources in the model. (Bakker & Demerouti, 2007)		
		competencies & abilities	All personal competencies and skills that facilitate working from home (also, for example, knowledge of how to use software).	“And that works quite well in most cases now. So this self-discipline is simply very much in demand, and I had to learn that myself first.” (interview #8)<br><br>“So I'm very fortunate, I'll say, in the use of my electronic tools, the programs that I have available and the software and so on, that I get on well with them and that I try to use them effectively for myself.” (interview #1)	
		working from home experience	Experience with working from home and therefore confident in working from home, skills already built up.	"This was a completely new way of working for some employees in our company." (Interview4)<br><br>"Of course, many of our colleagues have had ten years, 12 years, 13 years of experience. This means that we are a routine operation, since many of our colleagues are no longer in their offices at all, but work from home, because they are in charge of construction sites or are at construction meetings, this was actually very well trained." (Interview 7)	
		personal strategies	Strategies that employees use to work effectively at home, in a way that promotes health and makes proper use of their resources	"So, yes, there are different strategies, well um, if you're constantly online and constantly responding to your e-mails, you don't really you don't really get to work. You just have to find ways for yourself, maybe to structure it a bit." (Interview1)	
	Job Autonomy			“I think that's the higher self-determination. So I can, for example, what's a very big factor is taking breaks. So I really have a problem taking a break here in my office.” (interview #3)	
		time flexibility		"And in itself it is allowed that you do your work between 6:30 and 20:00 and the number of interruptions and the duration of the interruptions in itself do not matter at all." (interview #6)<br><br>“The additional flexibility gained (..), but essentially limited to the fact that there was simply more time available due to the elimination of commuting.” (interview #8)<br><br>"Let's put it this way, greater freedom to organize one's day. Yes, that's true. Just because there are no more commuting times and you can be more flexible with breaks and so on. Yes, and also that, especially if you have a family, you can take care of little things in between" (Interview 10).	Gained commute time, but also freedom to take longer lunch breaks
		decision latitude		“The most obvious freedom is, of course, that of clothing. Of course, since we don't have any video calls with high-ranking ministers or anything like that (laughs), we all sit in front of the screen in our leisure jogging gear, I'd say, and that's just pleasant, you don't have to pay that much attention to it now, do you?” (interview #6)	
		scope of action		"Of course, that's also one of the advantages of a home office: if I do nothing one day and more the next, you don't notice that from one day to the next" (interview 7)	
	Work Task			"...we have task expansion and therefore, so this is an exciting environment."(Interview1)<br><br>"It has to be said quite clearly, yes. So the personal encounters. Yes, otherwise, that one works on current topics, yes, these are the aspects." (interview #10)<br><br>“Apart from that, of course, you can sometimes enjoy sitting at your desk and working on a translation with a lot of fine-tuning and in between doing a bit of terminology work, i.e. extracting terminology from the texts, etc. But it's often a bit much computer work for me.” (interview #5)	Work task itself is fun/satisfies and a resource

Theoretical Dimension	Main category	Sub-category	Definition	Quotes	Coding rules
	**Collaboration**		Working together with colleagues, even if changes or no changes are perceived.<br><br>All statements on cooperation between employees in a team, unit or agency also: duration of cooperation, intensity of cooperation and quality,	“It works quite well, so we don't notice any loss of performance or friction in the form of operational failures. But it's just a different way of working.” (interview #6)<br><br>“the team spirit is certainly not as strong as it was before” (interview #7),	
		Co-worker support	e.g. help with work tasks or setting up the software with colleagues at home	"But the ability to use technology to bring colleagues into a conversation virtually at the push of a button" (Interview 12)<br><br>"We have flat hierarchies, a friendly atmosphere and are always there for each other." (Interview #4)	
		social exchange	everything that has to do with communication, agreements and exchange among colleagues<br><br>communicative meeting of employees also: quality and frequency of communication	"On the other hand, talking to colleagues once in a while, this unplanned communication." (interview #8)<br><br>"(...) you have to think about something else from time to time, not just about, I don't know, what you're just working on. So it's most effective to do it outside and then just, whoops, see what's going on, is there someone there, have a quick chat, "And what's going on?" and then after ten minutes it's all over and you might have new ideas." (interview #2)<br><br>"And personally, the days that I'm on site, I'm always really happy to be there. Because I see the people, because I can exchange ideas with them, even have a longer conversation or two, and yes, I'm actually really happy to be there as a presence." (interview #9)<br><br>“These are the things that are missing, or colleagues from other areas that you meet and talk to, or that you arrange to meet for lunch, or things like that. These things are missing.” (interview #10)<br><br>"Of course, it's not the same. As far as the intensity is concerned and also as far as meetings are concerned, you can of course do it that way, but if it runs exclusively via distance communication, then the quality also suffers, yes." (Interview #10)<br><br>“So except for the communication thing a little more direct, straighter. By not leaving things and waiting until you can look for someone, but doing it immediately, then you have it out of your head. That's what (four? many?) colleagues did and I thought that was a real improvement.” (interview #7)<br><br>“…and then when they do call, they even apologize for calling "Sorry to bother you," where I then think: Yes, but I'm on duty. If I was sitting in the room next to you right now, they would also come in and just ask.” (interview #3)	social exchange with supervisor is coded with category leadership
		appreciation	Appreciation of one's own work by colleagues, customers (also feedback)	“But as a rule they are all very positive, not just towards me (laughs very lightly), I think, but it's just such good practice that most people say thank you when you've done something for them and at conferences or interpreting appointments, of whatever kind, this of course comes into play even more, because the participants in these conferences also notice how difficult it often is with the sound quality at such events.” (interview #5)	appreciation by supervisor is coded with category leadership
		team culture		"...and on top of that we have such a grassroots democratic (laughs lightly) team. Of course, theoretically someone can come along and say, "This has to be finished by ten," but we're actually in a different mood here." (Interview #5)	
	**Leadership**		Influence of managers on employee behavior through praise, feedback, social exchange...	"So mostly, so how it is in a public agency, or in any other place outside the public sector, the orders come from the superiors the orders come and in the other direction the results are reported." (interview #1)<br><br>"Quite the opposite: If the technology displays it well, then everything is discreet. No one has to worry: Oh dear, if I go into the boss's office in the long hallway, the person sitting across from me in the office will see that. No one will see who I'm sitting with in the WebEx meeting." (interview #12)	
		appreciation	Appreciation of one's own work by supervisor	“It works, it works well. So you also receive praise or whatever, or personal interest, and I can't complain about that this year. But it has also been different. With other superiors before that, it was totally different, and that's something that was also a burden for me.”(interview #1)	

Theoretical Dimension	Main category	Sub-category	Definition	Quotes	Coding rules
		Supportive attitude of leader towards working from home (enabling working from home)		"Yes, I first had to come to terms with communication and this question of trust, so I first had to learn that. But apart from that, I have actually had relatively few problems." (interview #11)<br><br>“I think that was not allowed. I don't know, by now it might be allowed, I don't know. Anyway, when working from home started due to Corona, I told them to "Take the monitors home" so that they can work properly.” (interview #2)<br><br>“But apart from that, it's a great deal of trust that the (name of agency) places in you (...) there is no kind of social control or somehow/ because everyone does it, it's the same situation for everyone, so no one says, "Yes, you sit at home and put your feet up," right?” (interview #6)<br><br>"For me, the most striking result was actually my attitude towards (working from home). It wasn't negative at the beginning, I don't want to say that, but I always had the feeling that I had to, I didn't want to control, but I always had the feeling: Does everyone work? Does everyone work the way they should work? Until this realization: That's not what it's about. It's about the result. And I can measure the result." (interview #11)<br><br>"And the flexible work is the next step, which means the work is done and not the presence is rewarded. That means we don't need the presence of people that I can control. If I control that, then I've made my first mistake as a leader, because presence says nothing. (...) So no better control through presence at the workplace, none." (interview #7)	
		social exchange with supervisor		“tries to talk to each of us at least once a day, right? That actually works out quite well. It's rare that that doesn't happen for a day.” (interview #6)<br><br>"I now have a supervisor, (...) who is very reserved in his communication. So if he calls me maybe once a month or twice, that's already a lot." (interview #11)	
	**Offers by agency**		Any agency action that supports working from home (equipment subsidy, online sports programs, etc.)	“(...) we have relatively many offers from the (name of agency), like so, there are (..) lunch break impulses or somehow from (name of agency), also active lunch breaks. I have all of those not at all used, but I could imagine that perhaps this has also helped other employees so a bit, yes not necessarily structuring, but perhaps that thereby contact was also once again established to other people.” (interview #9)<br><br>"By the way, our agencny has also introduced the idea that we have a total of 25 hours free per year that you can spend on health management. That means you can take half an hour or an hour, but you shouldn't take it all at once and then you can say, "Yes, I went for a walk, or I went cycling," and the quota you're allowed to spend per year is 25 hours." (interview #2)<br><br>"You can sign up for online yoga courses, for example, or there are nutritional tips once a week with a recipe suggestion, and so on. The office also takes care of the employees and tries to actively focus on employee satisfaction and employee health." (interview #6)<br><br>"First of all, all people had to have a chance to decide for one or the other (laptop or Ipad and Iphone). Then it was "That's it. What you order now, that's what you'll get." Then it was delivered at some point (...)" (interview #5)<br><br>"We are currently in talks with the agency to see if there are any subsidies for office furniture. That's why (laughs very lightly) we're holding back on private purchases at the moment." (interview #6)<br><br>"That has actually changed a bit. That was/ In the meantime, that has been a topic in many rounds, also leading at a distance et cetera. We now have extra workshops on this matter. How to deal with it" (interview #8)<br><br>"And they do all the leadership training. So every manager has to do this training. That's four or five courses, except this digital communication and leading at a distance, that's new, they don't know that." (interview #11)	

Theoretical Dimension	Main category	Sub-category	Definition	Quotes	Coding rules
	**Work Environment and (technical) equipment at home**		Workplace equipment in technical and spatial terms (e. g. software for collaboration)<br>Working environment	“And that is also sacred to me. I set that up for myself when I first started telecommuting, which was early 2019. It's a room of its own. I built a huge desk myself that goes through the entire length of the room. Then I have a private workstation there, my official IT workstation. Here I also have absolute peace and quiet, and I enjoy that. And I can close the room. So then, I can't see what's there.” (interview #11)<br><br>"If you yourself live in a spatial situation that is more affine to single-family homes or live on the outskirts of town - when I open the door, there's something green, there's fresh air - then it's different, isn't it?" (interview #12)<br><br>"What is, however, I also have two large monitors here in the home office plus the monitor from the laptop. That means I work with three monitors and my colleagues do the same." (interview #2)<br><br>"I have to say that I use some private equipment when I work from home. A second monitor, for example." (interview #1)<br><br>“We had some laptops, but not all, so that at the beginning the laptops had to be exchanged in part. So one person got the laptop for a week and the other had to find something else to do at home, which was difficult.” (interview #11)	
**Job Demands**			Job demands refer to those physical, psychological, social, or organizational aspects of the job that require sustained physical and/or psychological (cognitive and emotional) effort or skills and are therefore associated with certain physiological and/or psychological costs. (Bakker & Demerouti, 2007)<br><br>Examples are a high work pressure, an unfavorable physical environment, and emotionally demanding interactions with clients. (Bakker & Demerouti, 2007)		
	**Physical demands**		frequent business trips, a lot of sitting, lack of exercise	"Except that I don't sit in the car as long anymore, no" (Interview 4)<br><br>"I think that's a good thing, because many events that would otherwise have required long journeys are now being held by video conference." (interview #3)	
		lack of excercise		“ (...) when I notice that I'm a bit sedentary, because I don't have to travel to the bus stop in the morning, I don't have to walk from the bus stop to the workplace, so those are all steps that are no longer necessary.” (interview #9)	
		ergonomy	demands such as long sitting activities<br><br>e. g. back complaints due to lack of workstation equipment when working from home	"Now in the online situation, some of the appointments follow each other seamlessly and I just click a link from one meeting to the next and that is, as I said, without a break. Sometimes, I don't even get up, then one appointment is over at ten, the other one starts at ten..." (interview #8).<br><br>“In the beginning, I moved in between, and in the beginning I kind of worked at the kitchen table, and then I got really bad back pain, but I've had a standing desk for four months now, and since then it's been okay again.” (interview #9)	
	**Psychological demands**				
		work delimitation		"This dissociation between private life and working life. That this must be regulated in some form and must also be scaled back from this current state." (Interview8)<br><br>"And that's already difficult, it's all mixed up here now. From morning to night. The business and the private. And that's something you have to get used to." (interview #1)<br><br>“(...) and in the evening also to find an end, because by this sluggish beginning I have determined personally for me, just this one time or in the evening at 10 o'clock turning the computer on is very tempting.” (interview #8)	

Theoretical Dimension	Main category	Sub-category	Definition	Quotes	Coding rules
		lack of social contact/ isolation		“If one has not seen then some colleagues perhaps (about a half year?) and then the first impacts came, where one has overheard things that a colleague has quit or has retired, the other has changed also the ministry, has quit or has oriented himself elsewhere...” (interview #6)	
		work-related availability		"I sometimes get emails, now from the [AGENCY] directorate, for example, that are sent at 11 p.m. or on Sundays, and I sometimes think to myself, "What's the point of that? I have the feeling that it is very important for them to show that "we are actually always available". Whether that's the case or not remains to be seen. But that's not the case in my area, no. (Interview11)	
		extended work-related availability		“I think it's more that I put myself under a bit of pressure, that I want to be available all the time (laughs lightly) because I want to show that I'm doing well. But I don't think the superiors expect that at all, yes.” (interview #9)	
		availability		“There is now an expectation that you are always there (available on the phone) from 8 a.m. to 6 p.m., as it were.” (interview #9)	
		enhanced stress due to digital communication	e. g. due to digital meetings without breaks inbetween	"Now in the online situation, some of the appointments follow each other seamlessly and I just click a link from one meeting to the next and that is, as I said, without a break. Sometimes, I don't even get up, then one appointment is over at ten, the other one starts at ten. We used to have ten-minute, quarter-hour, half-hour breaks in between. That's all gone now. So the calendar is much more tightly scheduled and with it the pressure to make the appropriate appointments, and that was actually not so stressful when I was there." (Interview8)	
	**Social demands**				
		in the office	Disturbances and interruptions of work by colleagues	"When you are under pressure to meet deadlines and you are in the same room as the others, there are always situations where you can't escape as quickly as you would in a home office. That can be exhausting." (interview #12)<br><br>“Then the next colleague comes and says "Someone just called. I couldn't tell you exactly either. Can you help me?” So then you're always out of your processes.” (interview #3)	
		working from home	Disturbances, demands by family members	"But (laughs lightly), when he's on the phone or something, it's so loud that I can hear it through the door. So (laughs lightly) it simply can't be prevented." (interview #5)<br><br>"And she certainly had problems at home, because the living situation was cramped and then they were together with her sister in the same room and so on. When we had video conferences, she had to go into the living room. So there were a bit more cramped situations." (Interview2)	
		delegation of tasks/ onboarding new employees more difficult		"In this context, it will be much more difficult to train new colleagues because the contacts are simply fewer." (Interview8)<br><br>"Sometimes it is more difficult to delegate certain tasks, precisely because everything is done via remote communication and it is not possible to go one or two rooms away and briefly discuss the task face to face. That simply makes it a bit more tedious." (interview #10).	
		establishing relationships more difficult		“Small talk should not be underestimated. It's also important for the relationship.” (interview #3)	
	**Organizational demands**				
		planning of office days	e.g. making appointments with colleagues in the office, planning activities that can only be done in the office (easier filing, printer available, workstation must be booked)	“There are a few tasks that you can only do in the office. For example, signing off on invoices and the like, which we don't yet do electronically. But then we also arrange to meet sometimes.” (interview #5)	
		organization of work material/ lack of work equipment		“Yes, of course, when someone gets the notebook home and then has to look at a 14-inch monitor all the time, I think that wears you out. No, I'm quite sure of it: It gets on your nerves, and it also wears out your performance.” (interview #12)<br><br>“We had some laptops, but not everyone, so that at the beginning the laptops had to be exchanged in part. So one person got the laptop for a week and the other had to find something else to do at home, which was difficult.” (interview #11)<br><br>"But it turned out that there were problems because we didn't have access to our work computer. No? So all the e-mails that somehow reached me, I couldn't answer them, I couldn't see them at all, and phone calls couldn't be diverted to my cell phone or my home phone, so there were already restrictions, ne, on how you could act." (interview #3)<br><br>"That was, yes, technical access to certain documents was simply not possible." (interview #8)<br><br>"What I find a bit more difficult when I work from home than when I'm in the office is the whole situation with printers and so on. I have a home printer here, which of course doesn't have anywhere near the performance that a nice copy machine like this has in the office." (interview #2)	

Theoretical Dimension	Main category	Sub-category	Definition	Quotes	Coding rules
**Health**					
	**physical health**				
		excercise		"Because I simply do some exercise at lunchtime and I somehow have the feeling that after lunch or after a lunch break, my brain switches off automatically, which is a bit of an exaggeration, but when I work from home I really start somehow fresher into the second half of the work day after lunch break." (interview #9)<br><br>"Because many have found the excercise is missing, the physical tension increases and the social exchange is also not there." (interview #8)	
		ergonomy		"But apart from that, I fortunately don't notice anything in terms of health, i.e. as far as movement restrictions are concerned. Of course, it's always the case with the wrong chairs, the wrong furniture, the wrong chairs, the wrong posture, that at some point you get movement problems (...)" (interview #6)	
		food intake		"So at lunchtime, that one cooks for oneself or cooks in larger communities, that has with the contact ban to do, happens now rather not. So meals tend to take place in isolation. That's stupid. That's really stupid, isn't it? You have to say that so clearly. That's a cut, and then at some point n-tv doesn't help anymore if I watch the latest news (laughs slightly) while eating, which is also totally unhealthy." (interview #12)<br><br>"(...) because business travel diet consists of curried sausage and hamburger and, yes, serving fries or something like that in the hotel. But now when you're at home like that, you can make yourself a cauliflower soup and make yourself a salad or something like that and eat, I think, healthier." (interview #2)	
		less cold symptoms		"(...) that someone coughs on you or something, that all falls away. So I'm actually/ no, I haven't had a cold at all during all this time." (interview #5)	
	**mental health**				
		stress		"Not that I would feel mentally ill now, but that's just this stress factor, you know?<br>This feeling of stress is much lower when I work from home. This stress factor. And that overall, of course, has a positive effect on my overall health situation." (interview #3)<br><br>"Because for the colleagues the stress, the stress of commuting and the stress of being annoyed every morning about train things and the devil knows what all, the long travel time or traffic jam or whatever, falls away. And nobody can tell me that standing in a traffic jam for hours in the morning isn't stressful."(interview #7)	
		exhaustion		"I think I feel much fitter since I started working from home. I mean mentally. So that means this exhaustion that I sometimes had when I was at work [at the agency] for a long time." (interview #11)	
		relaxation		"Of course I also have appointments when I work from home, but I can better take breaks, relax better in between or maybe even go for a walk around the block than when I work on site." (interview #3)	
		quality of life		"The fact that I can organize my everyday life so flexibly means that I no longer have to travel to work. So I have a travel time of over three hours in total, so one and a half hours each way, if not two. I would say yes, totally. So I have more quality of life because of it, much more time to do things that would otherwise simply be not feasible." (interview #9)	
		frustration/ dissatisfaction COVID-19		"But I know from others who have a lot to chew on, the social contacts, in between just being able to laugh with others or the like. That puts a damper on the mood. Fortunately, that's not the case for me personally." (interview #8)<br><br>And another point is that you can get a certain amount of cabin fever. That is, when you simply don't get out enough, don't meet enough other people, and move almost exclusively within your own family. That is also something that can lead to a certain dissatisfaction if it takes place over a longer period of several weeks. (interview #10)	
